# Patient-reported outcome measures are associated with health care utilization in patients with transplant ineligible multiple myeloma: a population-based study

**DOI:** 10.1038/s41408-021-00602-4

**Published:** 2022-01-27

**Authors:** Hira Mian, Rinku Sutradhar, Gregory R. Pond, Branavan Sivapathasundaram, Jonathan Sussman, Amaris Balitsky, Anita D’Souza, Tanya M. Wildes, Hsien Seow

**Affiliations:** 1 Department of Oncology, McMaster University, Hamilton, ON Canada; 2 Division of Biostatistics, DLSPH, University of Toronto, Toronto, ON Canada; 3 ICES, Toronto, ON Canada; 4 Division of Hematology/Oncology, Medical College of Wisconsin, Milwaukee, WI USA; 5 Cancer and Aging Research Group, St. Louis, MO USA

**Keywords:** Quality of life, Myeloma

Multiple myeloma (MM), a cancer caused by malignant plasma cells, is associated with morbidity and mortality. It is a disease of older adults with the majority of patients not receiving an autologous stem cell transplant [1]. Health care utilization is known to be high among patients with MM, leading to a significant treatment burden [2]. Undetected symptoms by health care teams and missed opportunities for the subsequent management of those symptoms may represent one cause of increased health care utilization in oncology [3, 4]. Symptom monitoring using patient-reported outcome measures (PROMs) is a strategy for detecting symptoms and conveying them to health care teams. Databases within Ontario, Canada represent a unique opportunity to evaluate the association of PROMs with health care utilization due to the implementation of a standardized population-wide PROM (the Edmonton Symptoms Assessment System [ESAS]) since 2007. The ESAS is a validated measure that assesses nine symptoms: pain, tiredness, drowsiness, nausea, lack of appetite, shortness of breath, anxiety, depression, and impaired well-being [5]. Patients score these symptoms on a scale from 0 (no symptom) to 10 (worse symptoms possible). ESAS assessments are voluntarily completed by oncology patients during outpatient clinic appointments. Although ESAS scores are routinely collected, changes in clinical management based upon these symptom scores occur infrequently [6] highlighting the need to better understand the association of these symptoms with patient-centered outcomes. The objective of our study was to evaluate the association between a patient-reported measure of symptom burden (ESAS score) and the subsequent 14-day risk of emergency department visits and/or unplanned hospitalization (ED/hosp) among transplant-ineligible newly-diagnosed (NDMM) patients in the first year following diagnosis.

Administrative health care databases were linked using a unique encrypted patient identifier and analyzed at ICES (formerly known as the Institute for Clinical Evaluative Sciences). ICES is an independent, non-profit research institute whose legal status allows it to collect and analyze health care and demographic data without consent for health system evaluation and improvement. The study obtained ethics approval at McMaster University and followed ICES guidelines with regard to data confidentiality and privacy.

Adults (age ≥ 18) with MM (International Classification of Diseases for Oncology, 3rd Edition, histology code 9732) between January 2007 and December 2018 were identified. Transplant ineligible patients were defined as those who received treatment with one or more of the following drugs (includes all funded anti-MM agents): cyclophosphamide, melphalan, thalidomide, lenalidomide, or bortezomib but no transplant within 1 year of diagnosis. Only patients who received treatment and reported at least 1 ESAS within 1 year following diagnosis were included. The exposure was ESAS score. All ESAS assessments over the study period were collected. Both individual symptoms (scores from 0 to 10) and total ESAS score (additive score of the nine individual symptoms, ranging from 0 to 90) were evaluated.

The study outcome was at least one ED/hosp within 14 days of an ESAS assessment among transplant-ineligible patients within the first year following diagnosis. A 14-day observation window was chosen consistent with a previous study [7] as it was felt that an uncontrolled symptom during this time could be correlated with an ED/hosp visit. A logistic regression model was used to assess the association of ESAS score and subsequent 14-day ED/hosp. A generalized estimating equations approach was used to account for patient-level clustering. All results were reported as adjusted odds ratio (OR) with 95% confidence intervals (CI) and statistical significance defined as a *p*-value < 0.05. Analyses were conducted using a statistical analysis system (SAS version 9.4).

A total of 4610 transplant-ineligible NDMM patients were identified among which 1734 (37.6%) were excluded due to the lack of an ESAS assessment. A total of 2876 patients completing 17,373 ESAS assessments were included. Baseline characteristics of the included cohort have been previously described [8]. Characteristics of the excluded cohort (*n* = 1743) are outlined in Supplementary Table 1. Patients in the excluded cohort were older, more often lived in an urban geographic location, had a lower socioeconomic status, more myeloma-related end-organ damage, and were treated more often at non-teaching sites as compared to patients that were included in our study cohort. Additionally, as ESAS assessment was completed more routinely in later years, a higher proportion of patients in the excluded cohort were diagnosed in earlier years and therefore had lower utilization of novel drugs.

Our cohort used 1755 ED/hosp visits following ESAS assessments among 1172/2876 (40.8%) transplant-ineligible NDMM patients within one year following diagnosis. From the 1755 ED/hosp visits, a total of 1183 (67.4%) were ED visits without hospitalization, and 572 (32.6%) visits were unplanned hospitalizations. The proportion of patients with ED/hosp within 14 days of the ESAS symptoms assessments based upon the individual and total ESAS score are outlined in Fig. 1. There was an incremental increase in the proportion of patients presenting to the ED/hosp in the subsequent 14 days with increasing ESAS scores (higher score indicative of worse symptoms) for individual symptoms and total ESAS score.

**Fig. 1:**
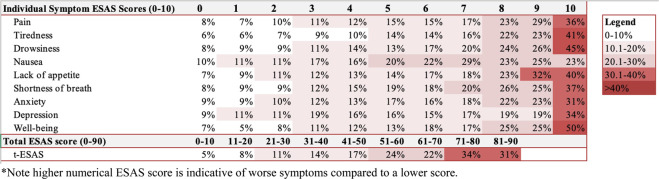
Proportion of patients in the cohort who presented to the emergency department/hospitalization within 14 days following symptom assessment stratified by the ESAS score*.

We conducted a multivariable regression analysis to examine the association of ESAS scores with 14-day ED/hosp (Table 1). Pain, tiredness, lack of appetite, shortness of breath, and impaired well-being were positively associated with 14-day ED/hosp, after controlling for other symptoms and covariates. Similarly, total ESAS score also was associated with the odds of ED/hosp noted to be 34% higher for every 10 unit increase in the score (OR = 1.34, 95% CI: 1.29–1.38, *p* < 0.01). Conversely, self-reported depression was the only symptom associated with decreased odds of ED/hosp (OR = 0.96 per unit increase, 95% Cl: 0.93–0.99, *p* = 0.01).

**Table 1:** Association between ESAS score and odds of emergency department /hospitalization within 14 days following symptom assessment.

OR^a^	Univariable			Multivariable^*^		
	OR	95% CI	*p*	OR	95% CI	*p*
*Individual ESAS symptom score (range: 0–10 each)*						
Pain	1.14	1.12–1.16	<0.01	1.06	1.04–1.08	<0.01
Tiredness	1.18	1.16–1.21	<0.01	1.05	1.02–1.08	<0.01
Drowsiness	1.15	1.13–1.17	<0.01	1.02	0.99–1.04	0.28
Nausea	1.13	1.10–1.16	<0.01	1.00	0.97–1.03	0.83
Lack of appetite	1.16	1.13–1.18	<0.01	1.07	1.05–1.09	<0.01
Shortness of breath	1.15	1.13–1.17	<0.01	1.07	1.04–1.09	<0.01
Depression	1.10	1.08–1.13	<0.01	0.96	0.93–0.99	0.01
Anxiety	1.12	1.10–1.14	<0.01	1.01	0.98–1.04	0.47
Well-being	1.18	1.15–1.20	<0.01	1.04	1.01–1.07	0.02
Total ESAS score (range: 0–90)^**^						
t-ESAS	1.37	1.33–1.42	<0.01	1.34	1.29–1.38	<0.01

^a^Odds ratio results are for one unit increase for individual symptoms and for a 10 unit increase for total ESAS score.

^*^Adjusted for other individual ESAS symptom scores, age, sex, geographic region, socioeconomic status, co-morbidity, treatment center, diagnosis year, anemia, hypercalcemia, renal failure, bone disease, novel drug usage, time from diagnosis to index ESAS, and receipt of treatment at index ESAS assessment.

**Not adjusted for individual scores given additive score.

In summary, our study characterizes the association of a PROM of symptom burden with health care utilization (14-day ED/hosp) in a population-based cohort of 2876 transplant-ineligible NDMM completing 17,373 assessments in the first year following diagnosis. Although several individual symptoms including pain, tiredness, lack of appetite, shortness of breath, and impaired well-being were identified in our study as being associated with an increased odds of experiencing a subsequent 14-day ED/hosp, depression was noted to have an inverse relationship. While the exact cause of this cannot be elucidated from our study, it is possible that depression may lead to social isolation and ineffective utilization of health care services [9]. As interventions for depressive symptoms are known to be poorly addressed [10], the finding from our study suggests the need to identify and proactively support interventions for patients reporting depressive symptoms.

Comparing our results to other research on PROM and its association with health care utilization is difficult given the paucity of data in this space, particularly among transplant-ineligible MM patients. Although benefits of PROM monitoring are well-established in oncology with randomized trials demonstrating improved symptom detection, patient quality of life, patient-clinician communication, and increased duration on chemotherapy in addition to improved overall survival [11, 12], there is a lack of data among real-world population studies. The few population-based studies which have evaluated this have focused on general cancer patient cohorts [13, 14] or specific cancers [7] and have not been reported in MM. A strength of our study is the use of real-world population data focusing on older patients who are often excluded from clinical trials [15]. Although we focused on patients who did not receive a transplant, patient–clinician preferences that led to the decision of not proceeding with a transplant cannot be elucidated from our study. Limitations include the lack of MM variables, such as stage, disease response, or the performance/frailty status, which could impact symptom burden and health care utilization [16].

In conclusion, our study establishes the association of PROMs with health care utilization among a large population cohort of transplant-ineligible NDMM patients. The results of this study may help MM patients, clinical care teams as well as health system administrators in identifying patients at high risk for ED/hosp. Furthermore, while not all unplanned health care utilization can be avoided, future research incorporating PROMs in risk prediction tools as well as prospective studies evaluating proactive symptom management among individuals at high risk for ED/hosp is needed to optimize this utilization.

## Supplementary information

Table S1
