# Supplementary material for: Patient-reported outcome measures are associated with health care utilization in patients with transplant ineligible multiple myeloma: a population-based study
Source: Blood Cancer J. 2022 Jan 27;12(1):17. doi: 10.1038/s41408-021-00602-4 (PMC8795114; doi:10.1038/s41408-021-00602-4)
Supplement: Supplementary file 1 — Table S1 [file 41408_2021_602_MOESM1_ESM.docx]

Table SI: Comparison of transplant ineligible NDMM patients included in the study cohort (completed ≥ 1 ESAS score ) and excluded (no recorded ESAS score during the first year after diagnosis)

| **Characteristics** | **Completed ≥ 1 ESAS score**  **(N=2876)** | **Completed no ESAS score**  **(N=1734)** | **P-value** |
| --- | --- | --- | --- |
| Age, years, median, IQR | 74 (70-80) | 76 (71-81) | <0.01 |
| Male (n, %) | 1649 (57.3) | 961 (55.4) | 0.20 |
| **Geographic Region, n (%)** |  |  | <0.01 |
| Urban | 2473 (86.0) | 1556 (89.7) |  |
| Rural | 403 (14.0) | 178 (10.3) |  |
| **Socioeconomic status, poor, n (%)** | 464 (16.1) | 464 (26.8) | <0.01 |
| **Charlson Co-morbidity Index, n (%)** |  |  | 0.52 |
| ≤ 1 | 2478 (86.2) | 1474 (85.0) |  |
| ≥ 2 | 398 (13.8) | 260 (15.0) |  |
| **Year of Diagnosis, n (%)** |  |  | <0.01 |
| 2007-2012 | 953 (33.1) | 945 (54.5) |  |
| 2013-2018 | 1923 (66.9) | 789(45.5) |  |
| **Myeloma end organ damage, n (%)** |  |  |  |
| Anemia | 911 (31.7) | 622 (33.3) | <0.01 |
| Hypercalcemia | 130 (4.5) | 106 (6.1) | 0.02 |
| Bone disease | 304 (10.6) | 228 (13.1) | <0.01 |
| Renal failure | 752 (26.2) | 566 (32.6) | <0.01 |
| **Hospital type, n (%)** |  |  | <0.01 |
| Teaching | 676 (23.5) | 295 (17.0) |  |
| Non-Teaching | 2200 (76.5) | 1439 (83.0) |  |
| **Novel agents, n (%)** |  |  | <0.01 |
| Immunomodulatory agent | 688 (23.9) | 311 (17.9) |  |
| Proteasome inhibitor | 2146 (74.6) | 1009 (58.2) |  |
